# Development of a Descriptive Profile and References for the Assessment of Taste and Mouthfeel Descriptors of Protected Designation of Origin Wines

**DOI:** 10.3390/foods11192970

**Published:** 2022-09-22

**Authors:** Anna Gomis-Bellmunt, Anna Claret, Anna Puig-Pujol, Francisco José Pérez-Elortondo, Luís Guerrero

**Affiliations:** 1 Catalan Institute of Vine and Wine (INCAVI), Plaça Àgora 2, 08720 Vilafranca del Penedès, Spain; 2 Food Quality and Technology, Institute of Agrifood Research and Technology (IRTA), Finca Camps i Armet, 17121 Monells, Spain; 3 Laboratorio de Análisis Sensorial Euskal Herriko Unibertsitatea (LASEHU), Lactiker Research Group, Universidad del País Vasco/Euskal Herriko Unibertsitatea (UPV/EHU), Centro de investigación Lascaray Ikergunea, Avenida Miguel de Unamuno 3, 01006 Vitoria-Gasteiz, Spain

**Keywords:** harmonization, accreditation, assessors’ selection, tasters training, TCA, official method, sensory analysis

## Abstract

Producers of PDO (Protected Designation of Origin) wines must submit to the EU authorities’ technical specifications that include the specific sensory description of each product typology, to be subsequently checked by the competent authority in each country. Unfortunately, there is no consensual and standardized approach for the development of sensory control methods for PDO wines. The aim of this work was to develop a sensory profile for the taste and mouthfeel descriptors that allows the characterization of wines from 11 existing PDOs in Catalonia (Spain), and with the purpose of advancing the process of harmonization of the official sensory analysis of wines. This paper includes the selection process of tasters, the procedure used for the definition and grouping of descriptors, and the development of references for the selected attributes. The use of this analytical tool should allow PDO/PGI product certification and control authorities to verify compliance with their specifications (descriptive and quantitative) based on objectively evaluated results.

## 1. Introduction

Wine is an ancient alcoholic beverage rooted in social, cultural, and economic life in many places across the world. The world’s surface area comprising vineyards is estimated to be 7.3 million hectares and the world’s global production of wine is 260 million hectoliters [1]. However, consumers’ behavior and the market strategies adopted are different in the so-called “Old World” countries (countries with a long tradition of production and consumption) and “New World” countries (more recent producers with limited consumption habits) [2,3]. The Old World countries protect, by legislation, the origin of the wine (associated with the geographical area of the vineyard) through the creation of figures such as the Protected Geographical Indication (PGI) or Protected Designation of Origin (PDO). New World countries have developed a differentiation strategy based on grape varieties [4]. However, as reported by Defrancesco et al. [4], there is an emerging debate on the appropriateness of this grape variety-based approach and the tendency of New World countries to introduce protected Geographical Indications (GIs) as additional quality signals linked to terroir. In fact, according to Josling [5], protected geographical indications can be a strategic tool for wine producers wishing to provide consumers with quality marks and influence their purchasing decisions. 

In general, consumers have a positive attitude toward products with collective quality labels, such as the Protected Designation of Origin (PDO) or Protected Geographical Indication (PGI), both linked to the origin of the product [6]. In the same vein, Grunert and Aachmann [7] also observed a favorable consumer attitude toward PDO-labeled products, who generally find them particularly attractive and evaluate them positively. With the aim of protecting consumers, European regulations seek to ensure that products labeled with a PDO, in addition to being products linked to a specific territory for their production and processing, offer a level of product quality, which must meet the physico-chemical and sensory characteristics specific of the area from which they originate. Thus, producers of PDO food products and wines must present EU authorities a technical specification of their products, which includes sensory descriptions according to Regulation (EU) 1308/2013 [8]. Moreover, the regulation establishes that the bodies in charge of controlling PDOs should be accredited in accordance with the ISO standard 17065 [9], and the sensory laboratories that analyze these products should be accredited in accordance with the ISO standard 17025 [10], which means the guarantee and demonstration of the technical competence of the laboratory, as well as the method used. Pérez-Elortondo et al. [11] analyzed the status of the implementation of this official sensory control and highlighted the need to harmonize a standard methodology for the sensory testing of PDO-labeled products. To comply with European regulations and to check whether a certain product (wine) satisfies the expected sensory characteristics, descriptive sensory analysis is essential. Both accredited sensory laboratories and tasting panels belonging to the PDO Regulatory Councils use their own method, which may or may not be similar to others. Thus, unfortunately, there is no consensual and standardized approach for the development of sensory control methods for PDO wines and, therefore, there is an evident need to harmonize the methodology, technical criteria, references, and appropriate lexicon to refer to each attribute analyzed [11,12,13]. 

Recently, Pérez-Elortondo and Zannoni [14] provided generic guidelines for the sensory analysis of PDO food products, including criteria and recommendations. In any case and regardless of the approach considered, an essential preliminary step is to describe the sensory characteristics and the use of appropriate terminology for the products to be controlled.

Descriptive sensory analysis has been applied to many products and has been studied by various authors [15,16,17], who agree that it is the most powerful tool for this purpose, since it allows both quantitative and qualitative aspects of the product to be addressed. The key point of this technique is the implementation process, which Murray et al. [15] referred to as a descriptive sensory program. This process includes the stages of selecting a panel to conduct sensory evaluations, the determination of a sensory language by which to describe product attributes, training the panel, and the validation of the panel to quantify the product attributes in a reliable way. Lawless and Heymann [17] summarized the implementation in three steps: training of the panelists, determining panelist performance during training, and evaluating the samples. The selection of panelists must be founded on factors such as commitment and motivation, availability, education, and the personality of the participants to be selected—factors that authors such as Guerrero [18] consider to be crucial, in addition to their sensory/physiological abilities. To this end, there are authors who have proposed the use of different initial tests to detect both the aptitudes and the motivation of judges [18,19]. Once the panel members have been selected, the next phase is the generation of the attributes or terms to be evaluated in the product, both using the existing nomenclature for the product when available and by generating the different terms to be assessed by means of the new panel [17]. The next step, concept formation, aims to consolidate the established lexicon and to harmonize its application so that all tasters can use it in the same way. This stage involves coming to a consensus of the intrinsic references of all tasters and adapting them to the product to be evaluated [15]. The most demanding part—especially in the world of wine, where there is extensive and idiosyncratic use of the sensory vocabulary—is likely agreeing on the objective meaning of each descriptor. Tasters should be actively involved in the whole process, so that their references are both qualitative (the presence or absence of a certain stimuli according to their individual threshold) and quantitative (points on the intensity scale) [15,20]. For this purpose, different intensity scales should be used to determine the suitability of each possible reference standard [17,21]. As stated by Rainey [22], reference standards are the best way to ensure that the scores given by a panel are objective and comparable. The last step when building a descriptive profile is to select and describe how to proceed with the analysis of the samples. Developing a common sensory methodology to evaluate any type of wine, regardless of its origin, is also a key point, since the way the product is prepared and tasted has a noticeable effect on the perceived sensory attributes and on their intensity [15,16,17,23].

The aim of this work was to develop a sensory descriptive profile that allows the characterization of wines from the 11 existing PDOs in Catalonia (Spain) in an objective and reliable way. Although the work has focused on the Catalan PDOs, it can serve as a reference guide in subsequent similar tasks, facilitating its implementation in new tasting panels and PDOs. This work also focuses on grouping under the same term, vague or even hedonic descriptors that sometimes appear in the specifications and hinder the harmonization process. Due to the larger number of attributes to be included, this paper focuses exclusively on taste and mouthfeel descriptors, with mouthfeel referring to sapid sensations activated by free nerve endings of the trigeminal nerve and taste meaning gustatory sensations detected by specialized epithelial receptor cells on the tongue [23]. To the best of our knowledge, this work is the first to simultaneously provide a detailed description of attribute selection, attribute reduction, and reference standard development for a large group of PDO wines (both qualitatively and quantitatively).

The whole document aims to help other labs and/or PDOs to develop and implement a sensory methodology, providing them with all of the relevant information to go a step further in the harmonization process of the sensory analysis of wines, in agreement with Pérez-Elortondo et al. [11].

## 2. Materials and Methods

### 2.1. Recruitment and Selection of Tasters

Candidates were recruited from tasters who were members of existing panels in Catalan PDOs. The call was extended to oenologists, sommeliers, and other professionals from the wine sector throughout Catalonia. A preliminary selection process was carried out by means of two sessions of three hours each, aimed at evaluating the candidates’ psychological and physiological aptitudes [18]. In addition, the candidates’ objective knowledge of wine was also obtained. For the visual acuity phase, the Ishihara test [24] and the online X-rite Color test IQ exercise [25] were performed. A scaling exercise was also carried out [26], as well as an odor and taste recognition test according to the ISO standard [27,28] and PROP status [29]. The specific selection consisted of four sessions of three hours each. During the first two sessions, the mean detection threshold of the group of candidates was determined for four different wine attributes (three olfactory and one gustatory), according to the method described by the International Olive Oil Council [30] adapted to wine. The olfactory attributes evaluated were 2,4,6-trichloroanisole (Sigma-Aldrich, Germany), blackberry aroma (SOSA, Barcelona, Spain), and 4-ethylphenol (Merck, Germany), while the evaluated taste attribute was acidity (citric acid solution). These attributes were selected according to their relevance in wine [31,32], their easiness of standardization, and the availability of information in the literature about them in terms of thresholds. Once the mean thresholds of the group of candidates were obtained, a specific screening test was carried out according to the intensity rating method for each of the four attributes [30] in two sessions, evaluating two attributes per session.

### 2.2. Selection of Taste and Mouthfeel Attributes

To select the attributes to be included in the sensory profile, the official technical specifications of the 11 Protected Designations of Origin (PDOs) included in the present study were examined. A total of 37 different wine typologies were identified, some of them common to several PDOs. Thus, combining wine typology and PDOs, a total of 114 wine types were obtained (e.g., Aged rosé wine PDO Catalunya and Aged rosé wine PDO Conca de Barberà) (Table 1).

The attributes used by each PDO to describe each type of wine were located in their corresponding sensory modality to allow ease of work (appearance, odor, flavor, taste, and mouthfeel). For each of the modalities, the original descriptors retained their original name from the official technical specifications of the PDO products and were summarized in a table, so that the rows contained the type of wine (the 37 typologies described in Table 1) and the columns contained the PDO of origin. This paper focuses only on the attributes of the taste and mouthfeel modalities.

To select the attributes to retain, three working sessions were carried out with the 30 tasters, each lasting three hours. In each session, the tasters were divided randomly into five groups of six people. The sessions were split into two parts. In the first part, each group had a summary table of all of the descriptors for the taste and mouthfeel modalities on a sheet of DIN A2 paper. They also had the technical specifications of all of the PDOs as support material. Then, they were asked to group the descriptors based on their perceived similarity, with the aim of identifying synonyms and unclear and subjective terms, and reducing the number of attributes to a practical and manageable level. After this, each group had to name each taste or mouthfeel based on their own group of attributes and had to try to define it. To perform this task, the tasters relied on their own sensory knowledge and personal experience. They also had additional information such as oenology books [23,33,34,35,36] and a laptop with internet access. In the second part of each session, an open discussion was held between the six groups, led by the panel leader. The discussion focused on reaching a consensus about the taste and mouthfeel descriptors to be retained, their definition, and the associated synonyms (e.g., rough or astringent) or subjective terms. It is important to note the necessity of maintaining the relationship between the name selected for a given attribute and its synonyms as, sometimes, these synonyms are the terms that appear in the technical specifications of the PDOs. In the last working session, two numerical formulas were defined to evaluate balance and chemical complexity. These two formulas were computed from the attributes already assessed. 

The attributes that were retained were sweetness, acidity, salty taste, astringency, structure, balance, chemical complexity, alcohol integration, and presence and integration of carbon dioxide. According to the demands of the technical specifications and the requested information by the different Regulatory Councils, sweetness, acidity, salty taste, astringency, and structure were assessed by means of a quantitative scale; meanwhile, balance, chemical complexity, alcohol integration, and presence and integration of carbon dioxide were assessed through qualitative variables (dichotomic or categorical).

### 2.3. Development of References

The development of references was performed in different steps. First, the main compounds that could potentially be used to represent each attribute and their concentrations to cover the usual range of intensities perceived in the wine [23,36,37,38] were identified. According to the database of the 11 PDOs involved in this study, the acidity of most of the wines ranged between 3.0 and 8.0 g/L, expressed as tartaric acid, and the concentration of total sugars (glucose + fructose) between 0.0 and 39.0 g/L. This information was considered as an indicator of the normal concentration ranges when preparing the sensory references for acidity and sweetness. It is worth mentioning that in the case of sweet wines, the sugar concentration can reach up to 150 g/L [39,40,41].

At the same time, the suitability of four possible matrices for adding the different compounds was qualitatively assessed. These matrices were aqueous, hydroalcoholic, and a synthetic wine with or without tannins. Table 2 shows the compounds that were evaluated for each attribute and the main characteristics of the different matrices after selecting the most promising ones by pretesting them with the panel. Then, for the quantitative descriptors, a combination of the different compounds and matrices was assessed for its intensity and suitability (similarity with the perception of the expected stimuli in a real sample). The intensity was scored on a 15 cm semi-structured linear scale anchored at the beginning of the scale with 0 (undetectable) and at 10 cm (maximum that can be found in a wine), leaving the possibility of scoring above 10 when the intensity of the sample was perceived as excessive. The suitability was measured on a semi-structured linear scale of 10 cm anchored in both extremes with 0 (not suitable at all) and 10 (totally suitable). The answer sheet contained the definition of the attribute and the terms (synonyms) that they included. The tasters could add any comments they considered appropriate. Based on the results obtained, three or four intensity points of the scale for each quantitative attribute were retained as reference standards. For each attribute, three evaluation sessions were conducted.

For the development of the references, the group of 30 tasters was divided into two groups of 15 participants each. The sessions lasted for two hours. Each session was divided into two parts; the first part involved individual sensory evaluation in tasting booths in a standardized sensory room according ISO Standard 8589 [42], while the second part comprised an open discussion carried out in a classroom equipped with a screen to display the results. The samples were presented monadically in 150 mL opaque white plastic cups, at a serving temperature of 20 ± 2 °C, in the same order for all the tasters and were identified with random three-digit codes. 

### 2.4. Statistical Analysis

To determine the intensity value for each quantitative reference and its suitability, a two-way ANOVA was performed that included the samples (different concentrations) and tasters as fixed factors. Tukey’s Honestly Significant Difference (HSD) post-hoc test was used to explore the existence of statistical differences among the concentration data (*p* < 0.05). All statistical analyses were performed using XLSTAT software, version 2020.1 (2020) (Addinsoft, Paris, France).

## 3. Results

### 3.1. Recruitment and Selection of Tasters

The initial group of candidates was made up of 96 people, of whom 81 participated in the preliminary and specific selections. Finally, the 30 individuals who obtained the best scores in the intensity rating test and did not present any remarkable physiological alterations were selected [43].

The detection thresholds for the 81 candidates in an aqueous solution were between 0.014 and 0.420 mg/L for 4-ethylphenol, 0.015 and 0.480 g/L for citric acid, 0.000125 and 0.008 mL/L for blackberry aroma, and 1 and 55 ng/L for 2,4,6-trichloroanisole. The identification thresholds were between 0.014 and 1.680 mg/L for 4-ethylphenol, 0.030 and 0.480 g/L for citric acid, 0.000125 and 0.008 mL/L for blackberry aroma, and 4 and 64 ng/L for 2,4,6-trichloroanisole. The final detection thresholds retained for the intensity classification method [30] were 0.097 mg/L for 4-ethylphenol, 0.0378 g/L for citric acid, 0.00264 mL/L for blackberry aroma, and 36.63 ng/L for 2,4,6-trichloroanisole. Based on this method, 3 (4%), 4 (5%), 3 (4%), and 33 (41%) candidates did not pass the test for 4-ethylphenol, citric acid, blackberry aroma, and 2,4,6-trichloroanisole, respectively. 

### 3.2. Taste and Mouthfeel Attributes

Table 3 shows the attributes selected for the taste and mouthfeel profiles. In addition, the table contains the definition of each descriptor, as well as other associated terms and the type of wine and PDO in which it was mentioned. Terms referring to attributes such as acidity, astringency, structure, and balance were common in most wine typologies, with percentages of mention exceeding 35%. On the contrary, attributes such as sweetness, CO_2_ presence and integration, alcohol integration, chemical complexity, and saltiness were only mentioned in a limited number of wines (17%, 11%, 9%, 4%, and 1%, respectively). Most of these attributes refer to wines having distinctive sensory characteristics (e.g., saltiness or sweetness) that normally describe only a few wine typologies, such as sweet wines. In the case of chemical complexity and balance, the definition also includes how to compute the final score of the attribute. 

### 3.3. Sensory References

Table 4 summarizes the selected compounds, final concentrations for each reference point and attribute, and the most appropriate matrix to be used for each of them. In all cases, three points of the reference scales were identified (low-, medium-, and high-intensity), except for sweetness, whereby four different intensity points were retained. There were significant differences (*p* < 0.05) between the different points of the scale for all of the quantitative attributes. For the qualitative variables, the different figures refer to the description of each level. Synthetic wine, with (SWT) or without tannins (SW), was the most suitable matrix for all of the attributes, with the sole exception of acidity. In the case of acidity, the hydroalcoholic solution was scored as the most appropriate. All of the selected compounds and matrices were those who obtained the highest mean values in the suitability scale. In all cases, these mean values were higher than 6. 

## 4. Discussion

### 4.1. Recruitment and Selection of Tasters

The recruitment was carried out via professional associations linked to the wine sector. Most of the interested candidates were experts from the wine sector according to the definition provided by ISO Standard 5492 [44]. All of the candidates had previous experience with wine. As expected, they brought their own knowledge and contributed actively to the reference development process, and probably shortened the time needed for the whole process. On average, two tasting sessions were needed to develop an attribute. Their contribution also supported the subsequent training process and, as stated by Lawless and Heymann [17] and Gawel et al. [45], made the learning process of the references straightforward.

In the preliminary selection process, we considered the physiological and psychological traits of the candidates, as recommended by several authors [17,18,28,46,47]. According to them, exploring and assessing the personality characteristics of the candidates should improve the selection process and should facilitate subsequent group activity. In this vein, we were able to detect 15 candidates with problems of availability or lack of interest, who excluded themselves in the next planned sessions. Regarding the sensory skills (e.g., descriptive and discriminatory ability) of the candidates, two persons with daltonism and one more with low taste sensitivity and a reduced identification ability were excluded. 

Subsequently, the specific selection method [30] showed that the attribute in which most tasters failed (41%) was in the quantification and sorting of the samples with 2,4,6-trichloroanisole (TCA). This compound, in addition to having a low sensory threshold (4.6–5 ng/L in water, 6.7–10 ng/L in dry white wine, and 7.1 ng/L in dry red wine according to Cravero et al. [48] and Juanola et al. [31]), is a potent suppressor of olfactory signal transduction at low concentrations [49,50] and normally causes panelist fatigue [48]. Despite these problems, in our opinion, including TCA in a selection process is a wise decision considering that this compound is present in more than 80% of tainted or spoiled wine, champagne, and spirit samples collected from producers and returned bottles [31]. In our case, the starting threshold used in the specific selection method was 36.63 ng/L, much higher than that reported in the literature. This high value, theoretically even easier to detect, might have had the opposite effect by increasing suppression and fatigue, thus leading to lower panelist performance. It is worth underlining the high sensory threshold obtained for this compound. The only plausible explanation is the use of high concentrations of TCA during the threshold determination (between 4 and 64 ng/L), which again might have induced suppression and fatigue in the panelists. This is an important issue to consider in future studies. This attribute was decisive in determining whether a taster was selected or not. Regarding 4-ethylphenol, the obtained detection threshold (0.097 mg/L) was lower than that reported in the literature (0.130 mg/L in water, 0.440 mg/L in aqueous alcoholic solutions, and 0.605 mg/L in red wines) [51]. This fact seems to confirm that the use of expert tasters implies lower thresholds, in agreement with [32]. In the case of citric acid, the detection threshold was 0.0378 g/L, similar to those values found in other studies [52]. For this compound, the supposed advantage of using trained tasters (lower thresholds) was not observed, probably because the sensory thresholds for citric acid have low inter-individual variability, as suggested in the results obtained by Mojet et al. [53]. Finally, regarding blackberry aroma, the observed sensory threshold (0.00264 mL/L) cannot be compared with any previous published paper, since it was obtained from a commercial product.

### 4.2. Taste and Mouthfeel Profiles

Theoretically, a sensory profile should be sufficient to describe different products within the category of interest [54]. For this reason, significant efforts are usually made to select the appropriate descriptors to constitute the final sensory profile [26]. In our case, the descriptive lexicon of the product was contained in the approved technical specifications of the different PDOs; thus, it was not necessary to generate new terminology, in contrast to other authors [55,56,57,58,59]. Although, in some cases, these organoleptic descriptions contained generic, hedonic, and terms that cannot be measured objectively (e.g., happy, fresh, and powerful). In addition, the variability associated with the use of different terms to refer to the same descriptor makes it difficult to compare results between different studies [13]. For this reason, we selected, defined, and grouped the already existing descriptors in an attempt to establish a common term for similar attributes. As Pérez-Elortondo and Zannoni [14] highlighted, with inadequate organoleptic descriptions, it is very difficult, even impossible, to develop a useful sensory scorecard. In any case, and to comply with EU regulations [8,60], we could not eliminate (just redefined) any of the terms included in the organoleptic description of the wines listed in the technical specifications of the PDOs. These terms refer to the typicity of each wine and of the terroir, and all of them must be assessed to certify a product by comparing its sensory properties to the technical specifications.

The final selected attributes (sweetness, acidity, salty sensation, astringency, structure, alcohol integration, and presence and integration of carbon dioxide) are all common attributes found in the literature for wines [23,37,57,61]. According to Flanzy [33], alcohol integration (warmness) and the presence and integration of carbon dioxide gas can be regarded as tactile sensations, and for this reason, were included as elements of mouthfeel. Gawel et al. [55] and Pickering and Demiglio [56] also included the term warm (alcohol integration see Table 3) in their mouthfeel wheel. Other terms such as velvety, sour, fullness, volume, and body described by the same authors were also included in our definitions of the selected attributes (Table 3). It is worth mentioning that the terms fullness, volume, and body were included within the structure descriptor, in agreement with other authors such as Etaio et al. [62] and Laguna et al. [38]. However, other authors prefer to describe and use these terms individually [55,58].

To complete the final profile, two additional descriptors, balance and chemical complexity, were also computed. These two terms, not directly evaluated by the tasters, were calculated by the panel leader from the other quantitative measures taken individually. In the case of balance, the parameters considered were sweetness, acidity, and astringency, which are the parameters that most authors define as the main constituents of equilibrium (or balance) [33,36,63]. Taste complexity was calculated by taking into account the number of parameters present of the taste phase, i.e., a wine with a minimum of three quantitative taste attributes that have an intensity higher than 3 (Table 3) was considered complex. This calculation agrees with the definition of “chemical complexity” put forward by Spence et al. [64], which relates to the number of different compounds found in a specific product. However, and according to Tempère et al. [65], complexity is more than the simple addition of attributes; it is rather the possible interactions between them. In any case, our definition of complexity has nothing to do with the “perceived complexity” described by other authors [63,66,67], which refers to complexity as a subjective term, related to the quality of the wine and measured by means of questionnaires completed by consumers and/or expert tasters. 

### 4.3. Reference Development

#### 4.3.1. Quantitative References

A synthetic wine matrix was the most appropriate in all cases, as observed by Ferreira et al. [68] and Sáenz-Navajas et al. [57], with the sole exception of acid taste. According to these authors, additional improvements, such as the inclusion of glycerol, are also recommended, although, other authors [38] have stated that glycerol in concentrations present in wine does not influence the mouthfeel. In our case, we did not include glycerol to make the synthetic wine simpler and because the panelists did not consider it necessary. 

The sensation of acidity does not depend only on the total concentration of acids in the wine, but also on each particular type of acid [36]. Tartaric acid is the main acid present in wine [33,35,69] and is the most frequently used compound to reproduce an acidic taste [56,70]. In our case, the overall sensation produced by the individual acids did not resemble that generated by the wine. The most plausible explanation is the lack of interactions with the other components normally contained in wine [71,72], which were not present in the hydroalcoholic matrix, since they had been expressly eliminated to remove interference. Thus, the best results in our case were obtained with a mix of tartaric, malic, and lactic acids (highest mean value of the assessed suitability). 

Due to the existing wide range of sugar concentrations and wine typologies (from dry to sweet wines) [23,36,41], two scales of different intensities were initially proposed (one for dry wines and one for sweet wines). Finally, both scales were combined to achieve a simpler profile and a lower number of references to be memorized by the panelists. The use of a single-intensity scale with such a wide range, although simplifying the training process compared to the use of two different independent scales, implies an important loss of discriminant capacity, as within the dry wines, all have relatively low scores at the bottom of the scale. However, a narrower range would have implied greater precision in the use of the scale [73]. From a practical perspective, we opted for a single scale, since only the sweetness attribute is included as a typicity parameter in sweet wines, while it does not appear in the remaining wine typologies. In fact, and according to Hufnagel and Hofmann [61], the sugar concentration in regular wines is normally below or close to the sensory thresholds. Regarding the type of sugar to illustrate sweetness, sucrose is mostly used as the reference standard [56,74]. Although sucrose is not naturally present in wine, it can be added to certain types of sparkling wines [75]. All wines have residual sugars that have not been fermented, the most common being glucose and fructose [23,76]. Glucose and fructose, together with ethanol and glycerol, are responsible for the perception of a sweet taste in wines [36,77]. However, in our case, since neither glucose nor fructose individually or combined produced a sensory perception similar to that of wine, we decided to use rectified concentrated grape must (RCGM) because of its similarity to wine sweetness. RCGM is an uncaramelized product obtained by the partial dehydration of grape must. The addition of RCGM is a common practice in some fortified wines, as it provides a sweetness that integrates perfectly with wine [78], in agreement with what was experienced by our tasters.

With respect to astringency, we decided to work directly with grape tannins. Grape tannins, extracted from the skins and seeds of grapes during fermentation [79], are responsible for astringency due to their polyphenol content, which interacts with the proteins and glycoproteins in saliva [80,81]. Grape tannins are the main source of polyphenols, together with the hydrolyzed tannins of oak barrels [61] in the case of aged wines. Different commercial brands of oenotannins were tested (see Table 2), thus obtaining similar results to the astringency perception in wines in all cases. Other authors have examined in depth the impact of different types of polyphenols involved in the perception of astringency [38,61,80,81,82,83,84], thus providing relevant information about the sensory sensation of each isolated compound. However, and from a practical perspective, the use of commercial natural products is more convenient and ensures that most of the compounds causing astringency are included. Other authors such as Etaio et al. [62] have also used commercial tannins as a reference standard for astringency.

Some typical constituents of wine such as yeast proteins (mannoproteins) and polysaccharides seem to be involved in generating the perception of body [23,38,80,81], which was defined as equivalent to structure in our case. To illustrate this descriptor, different types of macromolecules such as mannoproteins, Arabic gum, and carboxymethylcellulose [56,85,86,87] were tested (see Table 2) directly from pure compounds or oenological preparations normally used in the tartaric stabilization of wines. Finally, a commercial oenological product that included mannoproteins and proanthocyanidic tannin (see Table 4) was selected due to its similarity with the body/structure sensation normally perceived in wines. In any case, as stated by Jackson [23], the lack of a consensus about the meaning of this sensory descriptor explains why little progress has been made in its study or in the most appropriate references to illustrate it.

Normally, a saline sensation is difficult to detect in the sensory analysis of wines, and when present, it is often very mild [36]. To obtain an adequate reference for saltiness in wines, sodium chloride was tested first. This compound is the reference option in most standards, thus including those dealing with the sensory analysis of wine [37]. According to Polaskova et al. [77], sodium chloride and potassium chloride are the chemical compound influencers of a salty perception of wines. In the same vein, De Loryn et al. [88] used sodium chloride-doped wines (0.5 and 1 g/L) to determine the perception threshold of a salty taste. Pickering and Demiglio [56] recommended 1.5 g/L of sodium chloride in an aqueous solution as a reference standard for a discrete sensation. In our case, the most suitable option was a mixture of 50% sodium chloride and 50% sodium bicarbonate, since it gave a salty taste closer to the natural salty perception in wines (7/10 of average suitability compared to a standard salty wine according to the 30 tasters on the panel).

#### 4.3.2. Qualitative References

In the case of the qualitative references, the matrix used was the same as in the quantitative descriptors, but improved by adding tannins (see Table 4) to make it more similar to wine.

In our case, the integration of alcohol was associated, by consensus, with the term warm. King and Heymann [86] also used the term “low hotness mouthfeel” to refer to warm, as opposed to “high hotness mouthfeel” (irritating and tingling) detected when the alcohol is causing a gustatory disequilibrium leading to unbalanced wines [89]. According to King and Heymann [86], low and high hotness mouthfeels can be referenced by respectively using 100 or 200 mL of grape spirit 50% *v*/*v* dissolved in 1 L of filtered water. In a more generic way, Pickering and Demiglio [56] described hotness using a 15% *v*/*v* water solution to represent this mouthfeel, while they defined a 13% *v*/*v* water solution as warm. Therefore, we simply prepared different synthetic wine solutions with different alcohol concentrations and evaluated them. The alcohol was considered integrated or not by consensus between the tasters. However, it is worth mentioning that a higher concentration of alcohol does not necessarily mean a lack of integration. Thus, in our case, the tasters assessed to what extent the added alcohol was perceived as something natural to the product or as something added that does not belong to the wine. According to our tasters, the overall sensation was similar to the one perceived when salt is added to a food product during the cooking process or added when the product is already cooked; the saltiness intensity can be the same, but the overall saltiness perception is quite different regarding equilibrium, balance, and sodium release during the chewing process.

The measurement of the perception of carbon dioxide is a rather difficult task, since it includes auditory, visual, nociceptive, and tactile stimuli [90]. In the case of sparkling wines, the effect of carbonation is defined as a chemesthetic sensation, including the stinging tingling of bubbles in the nose and mouth [89]. There are different methods to add CO_2_ to a liquid, from natural fermentation by adding sugar to a hydroalcoholic solution and letting it ferment [91], or using semi-industrial systems of continuous injection or by injecting the gas into a closed vessel under pressure [92]. This system, similar to domestic carbonation systems, increases the internal pressure and, therefore, the solubility of the CO_2_, thus being easy to use and having a low cost.

A correct integration of carbon dioxide normally implies a natural fermentation process, since sparkling wine is defined as a hydro-alcoholic supersaturated solution of carbonic gas during its fermentation [91,93]. This natural process requires at least nine months of aging (by law); therefore, we built our references by means of a domestic carbonation system. 

## 5. Conclusions

This work provides a detailed guide on the selection and training of tasters for evaluation of the taste and mouthfeel attributes of wines with PDOs, as well as information on how to group and simplify the attributes described in the technical specifications of the different PDOs in a simple and practical way. It also includes the description of the references developed, which can be very useful when creating similar panels and constitutes a further step toward the process of methodological harmonization. Although the descriptive profile described (taste and mouthfeel attributes) was developed for the 11 Catalan PDOs, it is easily applicable to other PDOs as they usually include attributes and terms similar to those described in this paper. It should be noted that the described procedure, instead of creating a specific profile for each PDO and wine typology, attempts to define a generic profile that can be used for all of them without losing the ability to discriminate between them.

With respect to the tasters, it should be noted that most of them were experts (oenologists, sommeliers, or product experts). This fact, which initially constituted an advantage in the initial phases of the selection, grouping, and definition of the terms to be included in the descriptive profile, as well as in the development of references (especially in the assessment of their suitability), could constitute a problem or bias in the subsequent evaluation of commercial samples. 

This analytical tool should enable PDO/PGI product certification and control authorities to verify compliance with their specifications (descriptive and quantitative) on the basis of the objectively evaluated results, thus providing a solution to the current needs of the wine sector.

## Figures and Tables

**Table 1:** Types of wines included from the technical specifications of the 11 Catalan Protected Designations of Origin.

	Wine Typology	Protected Designation of Origin
1	White wine	a, d, e, f, g, h, i, j, k
2	Young white wine	b, c
3	Low-alcoholic white wine	b
4	Aged white wine	b, c
5	White wine fermented in barrels on lees	e
6	White wine aged in wood	e, i
7	Rosé wine	a, d, e, f, g, h, i, j, k
8	Young rosé wine	b, c
9	Low-alcoholic rosé wine	b
10	Aged rosé wine	b, c
11	Rosé wine fermented in barrels on lees	e
12	Rosé wine aged in wood	e
13	Red wine	a, d, e, h, i, k
14	Young red wine	b, c, f, g, j
15	Low-alcoholic red wine	b
16	Aged red wine	b, c, f, g, j
17	Red wine fermented in barrels on lees	e
18	Red wine aged in wood	e, i
19	Quality sparkling wine	a, c, d, e, g, h, j
20	*Vi d’agulla* (sparkling wine)	a, b, c, d, e, g, h, j
21	Liqueur wine/fortified wine	a, b, c, d, g, k
22	Natural sweet wine	c, f, h, i, k
23	Sweet liqueur wine	i
24	*Ranci* wine (dessert wine with oxidative notes)	c, f, h, i, j, k
25	Sweet *Ranci* (sweet wine with oxidative notes)	i
26	*Mistela* (sweet wine)	c, h, j
27	White *mistela* (sweet wine)	e, f, i, k
28	Red *mistela* (sweet wine)	e, f, i, k
29	*Garnatxa* (sweet wine)	e, f, j
30	*Moscatell* (sweet wine)	e, j
31	Classic DO Tarragona (dessert wine)	j
32	Sacramental wine	j
33	Sweet wine	e
34	Late-harvest wine (from overripe grapes)	e, g, i
35	*Vimblanc* (sweet wine)	f, i, j
36	*Dolç de fred* (ice wine)	g
37	*Vi de finca* (single-vineyard wine)	i

a: PDO Alella; b: PDO Catalunya; c: PDO Conca de Barberà; d: PDO Costers del Segre; e: PDO Empordà; f: PDO Montsant; g: PDO Penedès; h: PDO Pla de Bages; i: PDO Priorat; j: PDO Tarragona; k: PDO Terra Alta.

**Table 2:** The compounds, concentrations, and matrices tested for each attribute.

Attribute	Compound	Concentration (g/L)	Matrix ^1^
Acidity	L(+)Tartaric Acid 99.5% (Panreac, Barcelona, Spain)	4.0, 5.0, 6.0, 7.0, 8.0	aq/HA
	Citric Acid 99.5% (Agrovin, Ciudad Real, Spain)	4.8, 6.0, 7.2, 8.4, 9.6	aq/HA
	DL-Malic Acid 99% (Panreac-AppliChem, Barcelona, Spain)	4.4, 5.4, 6.5, 7.6, 8.7	aq/HA
	MixAcid LM (AEB Ibérica, Barcelona, Spain) mix of lactic and malic acids	3.0, 4.0, 5.0, 6.0, 7.0, 8.0	HA
	MixAcid TL (AEB Ibérica, Barcelona, Spain) mix of tartaric and lactic acids	3.0, 4.0, 5.0, 6.0, 7.0, 8.0	HA
	MixAcid TM (AEB Ibérica, Barcelona, Spain) mix of tartaric and malic acids	3.0, 4.0, 5.0, 6.0, 7.0, 8.0	HA
	MixAcid TLM (AEB Ibérica, Barcelona, Spain) mix of tartaric, malic, and lactic acids	3.0, 4.0, 5.0, 6.0, 7.0, 8.0	HA
Sweetness	D(+)-Glucose (Panreac-AppliChem, Barcelona, Spain) D(+)-Glucose (Panreac-AppliChem, Barcelona, Spain)	1.6, 3.1, 4.7, 6.3, 7.8 15.6, 46.9, 78.1, 109.3, 156.3	aq/HA aq/HA
	D(-)-Fructose (Panreac-AppliChem, Barcelona, Spain) D(-)-Fructose (Panreac-AppliChem, Barcelona, Spain)	0.8, 1.7, 2.5, 3.3, 4.2 8.3, 25.0, 41.7, 58.3, 83.3	aq/HA aq/HA
	Rectified Concentrate Grape Must, 64.7° Brix, 874.10 g/L sugar (Concentrados Palleja, S.L., Tarragona, Spain) Rectified Concentrate Grape Must, 64.7° Brix, 874.10 g/L sugar (Concentrados Palleja, S.L., Tarragona, Spain)	1.0, 2.0, 3.0, 4.0, 5.0 10.0, 30.0, 50.0, 70.0, 100.0, 130.0	HA HA
	Rectified Concentrate Grape Must, 64.7° Brix, 874.10 g/L sugar (Concentrados Palleja, S.L., Tarragona, Spain)	3.0, 10.0, 30.0, 50.0, 85.0, 130.0	SW
Astringency	VR Grape tannin (Laffort España, Errenteria, Spain)	0.5, 2.2, 3.6, 5.0, 6.0	aq/HA
	Protan Raisin, tannin (AEB Ibérica, Barcelona, Spain)	0.5, 2.2, 3.6, 5.0, 6.0	aq/HA
	Protan Raisin, tannin (AEB Ibérica, Barcelona, Spain)	0.5, 2.2, 3.6, 5.0, 6.0	SW
	Protan Raisin, tannin (AEB Ibérica, Barcelona, Spain)	0.5, 2.2, 3.6, 5.0	SW
	Protan Raisin, tannin (AEB Ibérica, Barcelona, Spain)	0.5, 1.5, 2.2, 3.6	SW
Saltiness	NaCl (Sharlab, Spain)/NaHCO_3_ (Panreac, Barcelona, Spain)	0.25/0.25, 0.50/0.50, 1.0/1.0	SW
	NaCl (Sharlab, Spain)/NaHCO_3_ (Panreac, Barcelona, Spain)/Sodium L-Glutamate 1-hydrate (Panreac-AppliChem, Barcelona, Spain)	0.25/0.25/0.25, 0.50/0.50/0.50	SW
Structure	STABIVIN SP, Arabic gum (Laffort España, Errenteria, Spain)	1.2, 1.8, 2.2 (ml/L)	SW
	ARABINOL HC, Arabic gum (AEB Ibérica, Barcelona, Spain)	1.2, 1.8, 2.2 (ml/L)	SW
	MANNOSTAB, mannoprotein (Laffort España, Errenteria, Spain)	0.3, 0.5, 0.8	SW
	BATTONAGE BODY, mannoprotein (AEB Ibérica, Barcelona, Spain)	0.3, 0.5, 0.8	SW
	NEW CEL, carboximethylcelulose (AEB Ibérica, Barcelona, Spain)	2.0, 2.5, 3.0	SW
	OENOLEES, Polysaccharide (Laffort España, Errenteria, Spain)	0.3, 0.5, 0.8	SW
	MANNOSTAB, mannoprotein (Laffort España, Errenteria, Spain)	0.05, 0.1, 0.3, 0.5, 0.7	SWT
	MANNOSTAB, mannoprotein (Laffort España, Errenteria, Spain)	0.05, 0.2, 0.6, 1.0, 1.4	SWT
	ELEVAGE Sweet, mannoprotein and proantocianidic tannin (AEB Ibérica, Barcelona, Spain)	0.1, 0.2, 1.2, 2.8	SW
Alcohol integration	Ethanol 96.42% *v*/*v* (Alcoholes Monplet SA, Barcelona, Spain)	12% *v*/*v*, 15% *v*/*v*, 18% *v*/*v*	SWT
CO_2_ presence	CO_2_ (SodaStream Iberia, Madrid, Spain) ^2^	1, 2, 3 pushes on the carbonating button	SWT
CO_2_ integration	CO_2_ (SodaStream Iberia, Madrid, Spain) ^2^	2, 3, 4, 5 pushes on the carbonating button	SWT

^1^ aq = mineral water Font del Pla Nova (Santes Creus, Spain), pH = 7.74; HA: hydroalcoholic dissolution 12% *v*/*v*, using ethanol 96.42% *v*/*v* (Alcoholes Monplet SA, Barcelona, Spain); SW (synthetic wine): hydroalcoholic dissolution 12% *v*/*v*, total acidity of 4 ± 0.5 g/L expressed by tartaric acid, using 7.5 mL of MixAcid TLM (AEG Ibérica, Barcelona, Spain), and 1.7g/L of potassium bitartrate 99% (Panreac-AppliChem, Barcelona, Spain); SWT: hydroalcoholic dissolution of 12% *v*/*v*, total acidity of 4 ± 0.5 g/L expressed by tartaric acid, using 7.5 mL of MixAcid TLM (AEG Ibérica, Barcelona, Spain), 1.7g/L of potassium bitartrate 99% (Panreac-AppliChem, Barcelona, Spain), 2g/L of Rectified Concentrate Grape Must (64.7° Brix, 874.10 g/L of sugar, Concentrados Palleja, S.L., Tarragona, Spain), and 0.05 g/L of Tannin Protan Raisin, (AEB Ibérica, Barcelona, Spain). ^2^ Prepared in the original SodaStream bottle, left at 4 °C for 24 h. Carbon dioxide was added in different concentrations with the sparkling water maker SodaStream JET (SodaStream Iberia, Madrid, Spain) equipped with a CO_2_ cylinder.

**Table 3:** Selected attributes, definitions, associated terms, and PDOs to which the terms belong.

Attributes	Definition	Associated Terms and Codes of the PDO and Wine Typology that Contain Them ^1^
Acidity/sourness	Basic taste produced by diluted aqueous solutions of most acidic substances, e.g., citric, malic, and tartaric acid	Acid: g1, g7, g13, g19, g20, g34, h19, j2, j16, j20 Happy: j20 Fresh: a1, a7, a20, b3, b2, b7, b20, c2, c7, c19, e1, e7, e12, e11, f29, g1, g7, h13, h19, i1, i13, j2, j7, j14, j19, j20 Acid core: h1, h20
Astringency	Complex taste sensation accompanied by the concentration, tightness, and puckering of the skin or oral mucosa produced by substances such as tannins	Astringent: j16 Tannic: a13 Mature tannicity: f13 Unctuous: a21, b4, b21, c4, c21, c22, e5, f29, g34, i24, i25, j26, j29, j30, j35, j32, j31 Silky: f1, j4, j7, j14, j16 Smooth: b16, c16, g1, g7, g13, g19, g20, i1 Mellow/honeyed: a7 Velvety: j16 Creamy: c19, h19, j19 Tasty: b14, b15, c14 Tactile: k (all wines)
Balance/equilibrium	Absence of taste edges, determined by the difference between the intensity of sweetness and the average intensity of the astringency and acidity The result will be interpreted as: −1 ≤ value ≤ 1: Highly balanced/equilibrated −2 ≤ value ≤ 2: Medium balance/equilibrium −4 ≤ value ≤ 4: Unbalanced/low equilibrated	Harmony: j1 Nice: j19 Balanced: a13, a20, b7, b20, c19, c20, d (all wines), e1, e7, e5, e6, e11, e12, f22, f28, h13, i1, i6, i24, i25, j1, j7, j13, j16, j26, j29, j30, j35, j32, j31, k (all wines) Elegant: f7, h13 Fine/ refined: a13 Honest: a (all wines), e (all wines), k (all wines) Correct attack (good mouthfeel): i7 Proper evolution: i7
Chemical Complexity	Wine called complex when it has a minimum of three quantitative taste attributes with an intensity >3	Intense: j24 Complex: f22, f24, f35, j16
Presence and CO_2_ integration	Tactile mouth perception caused by the presence of bubbles	Presence of carbon dioxide: d20, j19, j20 Integration of carbon dioxide: h19, j19 Sparkling: j20 CO_2_ well integrated: h19, j19 Tactile sensation of carbon dioxide: h20 Tickling in the mouth: b20, c20, h19 Perceptible carbon dioxide: d20
Saltines	Salty-mouth sensation, produced by elements such as fluorine, silicium, iodine, bromine, boron, and manganese	Saltiness: k1
Structure	Sensation in the mouth in which all of the attributes or tactile sensations are added	Sumptuous: h7, h20 Round: b16, c16, e5, f13, j14 Body: f24, k (all wines) Volume: f1, h19 Full: e5 Light: b2, b3, b7, c2, c7, c14, h1, h20, j2, j7 Fleshy: e11 Width: f13, h20, j24 Very structured: b16, c16, h7, h13, h20, i6, i13, i22, i23, i27, i28, i34, i37 Strong: h24 Blunt: f24, j24 Powerful: a7, h7, h13, h20
Sweetness	Basic taste produced by diluted aqueous solutions of natural or synthetic substances, such as sucrose, dextrose, and aspartame	Sweet: f27, f28, f29, h22, h26, i22, i23, i27, i28, i34, j16, j20, k22, k26, k27, k28 Dry: f24, h24, j24 Gourmand: f1, f7 Honeyed: a7
Well-integrated alcohol/warm	Integration of alcohol: Warm sensation that is in balance with the other components Warmness: Thermal sensation in the mouth that does not burn	Integrated alcohol: a1, j16, j20, k21 Warm: a21, b21, c21, e33, f22, k21

^1^ Each combination of letters and numbers indicates in which PDO and wine typology the specific term is mentioned. a: PDO Alella; b: PDO Catalunya; c: PDO Conca de Barberà; d: PDO Costers del Segre; e: PDO Empordà; f: PDO Montsant; g: PDO Penedès; h: PDO Pla de Bages; i: PDO Priorat; j: PDO Tarragona; k: PDO Terra Alta. 1: White wine; 2: Young white wine; 3: Low-alcoholic white wine; 4: Aged white wine; 5: White wine fermented in barrels on lees; 6: White wine aged in wood; 7: Rosé wine; 8: Young rosé wine; 9: Low-alcoholic rosé wine; 10: Aged rosé wine; 11: Rosé wine fermented in barrels on lees; 12: Rosé wine aged in wood; 13: Red wine; 14: Young red wine; 15: Low-alcoholic red wine; 16: Aged red wine; 17: Red wine fermented in barrels on lees; 18: Red wine aged in wood; 19: Quality sparkling wine; 20: *Vi d’agulla* (sparkling wine); 21: Liqueur wine/fortified wine; 22: Natural sweet wine; 23: Sweet liqueur wine; 24: *Ranci* wine (dessert wine with oxidative notes); 25: Sweet *Ranci* (sweet wine with oxidative notes); 26: *Mistela* wine (sweet wine); 27: White *Mistela* wine (sweet wine); 28: Red *Mistela* wine (sweet wine); 29: *Garnatxa* wine (sweet wine); 30: *Moscatell* wine (sweet wine); 31: *Classic DO Tarragona* (dessert wine); 32: Sacramental wine; 33: Sweet wine; 34: Late-harvest wine; 35: *Vimblanc* (sweet wine); 36: *Dolç de fred* (ice wine); 37: *Vi de finca* (single-vineyard wines).

**Table 4:** Selected compounds, matrices, and concentrations and their corresponding intensity in the sensory scoring scale.

Attribute	Compound	Selected Concentrations Expressed in g/L or Categories for Qualitative Descriptors ^1^	Matrix ^3^
Acidity/sourness	MixAcid TLM (AEB Ibérica, Barcelona, Spain) mix of tartaric, malic, and lactic acids	3.0 (3), 5.0 (5), 8.0 (8) ^2^	HA
Sweetness	Rectified Concentrate Grape Must, 64.7° Brix, 874.10 g/L of sugar (Concentrados Pallejà, S.L., Tarragona Spain)	3.0 (1), 30.0 (5), 85.0 (7), 130.0 (9)	SW
Astringency	Protan Raisin, tannin (AEB Ibérica, Barcelona, Spain)	0.5 (3), 1.5 (5), 2.2 (7)	SW
Saltiness	NaCl (Sharlab, Barcelona, Spain)/NaHCO_3_ (Panreac-AppliChem, Barcelona, Spain)	0.25/0.25 (3), 0.50/0.50 (5), 1.0/1.0 (8)	SW
Structure	ELEVAGE Sweet (g/L), mannoprotein and proantocianidic tannin (AEB Ibérica, Barcelona, Spain)	0.1 (2), 1.2 (5), 2.8 (7)	SW
Alcohol integration	Ethanol 96.42% *v*/*v* (Alcoholes Monplet SA, Barcelona, Spain)	12% *v*/*v* (well integrated)/18% *v*/*v* (poorly integrated)	SWT
CO_2_ presence	CO_2_ (SodaStream Iberia, Madrid, Spain) ^4^	2 pushes (presence of CO_2_)	SWT
CO_2_ integration	CO_2_ (SodaStream Iberia, Madrid, Spain) ^4^	2 pushes (well integrated)/5 pushes (poorly integrated)	SWT

^1^ In brackets is the corresponding intensity or category (for qualitative attributes) in the reference scale. ^2^ Buffered with 1.7, 1.7, and 4 g/L of potassium bitartrate 99%, respectively (Panreac-AppliChem, Barcelona, Spain). ^3^ HA: hydroalcoholic dissolution 12% *v*/*v*, with ethanol 96.42% *v*/*v* (Alcoholes Monplet SA, Barcelona, Spain); SW (synthetic wine): hydroalcoholic dissolution 12% *v*/*v*, total acidity of 4 ± 0.5 g/L expressed by tartaric acid, using 7.5 mL of MixAcid TLM (AEG Ibérica, Barcelona, Spain) and 1.7g/L of potassium bitartrate 99% (Panreac-AppliChem, Barcelona, Spain); SWT: hydroalcoholic dissolution of 12% *v*/*v*, total acidity of 4 ± 0.5 g/L expressed by tartaric acid, using 7.5 mL of MixAcid TLM (AEG Ibérica, Barcelona, Spain), 1.7g/L of potassium bitartrate 99% (Panreac-AppliChem, Barcelona, Spain), 2g/L of Rectified Concentrate Grape Must (64.7° Brix, 874.10 g/L of sugar, Concentrados Palleja, S.L., Tarragona, Spain), and 0.05 g/L of Tannin Protan Raisin, (AEB Ibérica, Barcelona, Spain). ^4^ Prepared in the original SodaStream bottle, left at 4 °C for 24 h. Carbon dioxide was added in different concentrations with the sparkling water maker SodaStream JET (SodaStream Iberia, Madrid, Spain) equipped with a CO_2_ cylinder.

## Data Availability

The data presented in this study are available on request from the corresponding author.
